# Evaluation of the Proximate Composition of *Amsonia tabernaemontana* Walt. Seeds and Glyceride Oil

**DOI:** 10.3390/molecules30020408

**Published:** 2025-01-19

**Authors:** Olga Teneva, Zhana Petkova, Ana Dobreva, Anatoli Dzhurmanski, Ginka Antova

**Affiliations:** 1 Department of Chemical Technology, University of Plovdiv ‘Paisii Hilendarski’, 24 Tzar Assen Street, 4000 Plovdiv, Bulgaria; olga@uni-plovdiv.bg (O.T.); zhanapetkova@uni-plovdiv.bg (Z.P.); 2 Institute of Aromatic Plants, Agricultural Academy, 49 Osvobozhdenie Blvd., 6100 Kazanlak, Bulgaria; anadobreva@abv.bg (A.D.); anatoli630914@gmail.com (A.D.)

**Keywords:** *Amsonia tabernaemontana* Walt., proximate composition of seeds, glyceride oil, fatty acids, sterols, tocopherols, phospholipids

## Abstract

The genus Amsonia, a member of the Apocynaceae family, comprises plants with notable medicinal benefits. In 2022 and 2023, *Amsonia tabernaemontana* Walt. seeds introduced to Bulgaria were collected and analyzed. Given the limited information available on the chemical composition of *A. tabernaemontana*, this study aimed to evaluate the phytochemical profile of the plant seeds collected over two consecutive years. Although members of the genus Amsonia are not conventional oilseed crops, the glyceride oil content was 7.8% and 11.1% in the respective samples. The chemical composition was meticulously analyzed, revealing carbohydrates in the largest amounts (60.4% and 61.3%), with crude fibers at 18.3% and 24.8%, and protein content at 19.5% and 13.0%. The amounts of ash and moisture content were also quantified. Additionally, the fatty acids, sterols, tocopherols, and phospholipids of the seed oil were examined. β-Sitosterol emerged as the main component in both harvests. The total tocopherol content was relatively low (52.7 mg/kg vs. 20.0 mg/kg), with α-tocopherol being predominant. Phosphatidylcholine, phosphatidylinositol, and phosphatidylethanolamine were identified as the principal components of the phospholipid fraction. The fatty acid composition primarily included linoleic (61.0 and 61.2%) and oleic acids (28.7 and 28.6%).

## 1. Introduction

Recently, a growing focus has been on consuming products with functional properties. Although there is limited information regarding the composition of many medicinal plants, some are appealing and beneficial to human health as alternative sources of biologically active compounds. These plants exhibit curative and preventive potential. Medicinal plants hold significant importance for human health and are widely utilized in various industries, such as cosmetics, pharmaceuticals, and foods. Most of these plants possess functional properties, playing a crucial role in treating and preventing chronic diseases [1].

The Apocynaceae family is a large group of plants, encompassing approximately 400 genera and 4555 species. These plants are predominantly found in tropical and subtropical regions across the globe. Most members of this family are flowering plants, which can be herbaceous, woody vines, shrubs, or even trees. Many species within the family are known to be poisonous due to the presence of glycosides and alkaloids, with applications primarily in medicine [2].

The genus Amsonia, a member of the Apocynaceae family, is globally distributed with 16 recognized species and numerous subspecies occurring in North America [3]. Two of the prominently grown species of Amsonia include eastern bluestar (*Amsonia tabernaemontana*) and Arkansas bluestar (*A. hubrichtii*) [4]. *A. tabernaemontana* is a widespread species found throughout the temperate forests of the southeastern USA, identifiable by its glabrous elliptic leaves, flat stigmatic head, and pubescent corolla tube [5]. *A. tabernaemontana* Walt. is an attractive plant, often cultivated as an ornamental. It is grown in parts of Europe, such as Poland and Hungary, and serves both decorative and medicinal purposes, containing alkaloids that have beneficial effects in treating cardiovascular or cerebral diseases [6,7].

*A. tabernaemontana* thrives in the spring, characterized by olive-green leaves containing rutin at a concentration of about 4–5%. The flowering period lasts 4–6 weeks, typically from late spring to early summer, producing pastel blue blooms. This plant exhibits remarkable adaptability to temperate and cold climates [8].

*A. tabernaemontana* is analogous to the common periwinkle (*Vinca minor* L.), also from the Apocynaceae family, suggesting similar composition and biological functions. *V. minor* is highly regarded in medical therapy, attracting significant pharmaceutical interest [9]. Cultivated as a medicinal plant, it is used to prepare active substances that enhance brain blood circulation [10]. Some physicochemical characteristics of *V. minor* oil include a relative density (d^20^) of 0.92, a refractive index of 1.462, and an iodine value of 36% I_2_ [11]. The fatty acid composition comprises stearic, linoleic, and oleic acids [10].

Conversely, the chemical composition of the genus Amsonia, particularly its seeds and glyceride oil, remains underexplored in the literature. The nutrient composition of *A. tabernaemontana* seeds, including their lipid, protein, and carbohydrate content, as well as the presence of lipid-soluble components, such as tocopherols, sterols, phospholipids, and essential fatty acids, has not been previously analyzed.

Given the lack of information about the chemical composition of *A. tabernaemontana* seeds and glyceride oil, as well as the lipid-soluble compounds in the species, this study aims to characterize the seeds composition and properties of *A. tabernaemontana* and assess its potential as a food and medicinal plant.

## 2. Results

Under the conditions of the Kazanlak Valley, the plants exhibit robust development and form a substantial herbal mass. They attain a height of 124 cm, a width of 168 cm, and produce 22 stems with diameters of 10 mm each. The yield of fresh herbs reaches 1810 g, while the yield of fresh inflorescences amounts to 330 g.

Amsonia is distinguished by its rapid growth during the initial stages of vegetation. In the present conditions in Bulgaria, the flowering phase commences in mid-May, merely two weeks after the onset of development.

### 2.1. Chemical Composition of A. tabernaemontana Seeds

The total contents of proteins, carbohydrates, crude fibers, ash, and moisture, as well as the energy value of the seeds from *A. tabernaemontana*, had not been previously examined. In the current study, the chemical composition of the seeds harvested over two consecutive years (2022 and 2023) was investigated, and the results are presented in Table 1.

The species *A. tabernaemontana* Walt. is not a typical oil crop, and both harvests were characterized by relatively low levels of glyceride oil (7.8% vs. 11.1%). The results show variations between the two analyzed harvests in terms of the protein content, with the 2022 harvest being richer in proteins than the 2023 harvest (19.5% vs. 13.0%). The carbohydrate content was nearly identical in both years (approximately 61%), underscoring its essential role as an energy source. Consequently, the energy values of both harvests were comparable, which can be attributed to the similar carbohydrate levels. The crude fiber contents were found to be 18.3% and 24.8% in the respective years. A notable difference was observed in the ash content, which was twice as high in the 2023 harvest (7.4%) compared to the 2022 harvest (3.7%). The moisture content ranged from 7% to 9%, which is typical of most of the plant species.

### 2.2. Physicochemical Parameters of A. tabernaemontana Seed Oil

The measured values of the physicochemical properties, including the peroxide value, iodine value, and refractive index of the seed oils, are presented in Table 2.

The peroxide value is an indicator of the formation of primary oxidation products (peroxides and hydroperoxides), which can lead to the deterioration of oil quality. The peroxide value (PV) of the studied *A. tabernaemontana* seed oil indicates that no oxidation occurred in the oils. The PV of both harvests was nearly identical—5.45 meqO_2_/kg and 5.29 meqO_2_/kg. Currently, there are no data from other research groups regarding the peroxide value of *A. tabernaemontana* species. The iodine value (IV) was also examined, indicating the degree of unsaturation of the oils. The IV of the 2022 crop was higher than that of the 2023 crop (139.8 g I_2_/100 g vs. 134.4 g I_2_/100 g). This was probably due to the slight changes that occurred in the fatty acid composition of the examined glyceride oil. The refractive index was measured and a difference was noted between the analyzed seed oils (1.4825 and 1.4873, respectively).

### 2.3. Lipid-Soluble Components in A. tabernaemontana Seed Oil

Many plants are recognized for their health benefits, owing to their rich bioactive compound content. Diets incorporating lipid-rich oils exhibit a range of beneficial health-promoting effects. These effects include anti-inflammatory, antimicrobial, anticancer, hepatoprotective and immunomodulatory actions, as well as protective properties against conditions like diabetes and non-alcoholic fatty liver disease [12].

The lipid-soluble compounds of *A. tabernaemontana* seed oil comprises unsaponifiable matter, sterols, phospholipids, and tocopherols. All components were identified in both plant harvests. The results are presented in Table 3.

The results indicate a very high content of unsaponifiable matter (over 20%), including sterols, tocopherols, etc. The 2022 crop was richer in unsaponifiable substances compared to the 2023 crop (25.5% vs. 19.5%).

The total sterol content of *A. tabernaemontana* seed oil was not significantly different in the two consecutive years of examination, ranging from 1.4% (harvest in 2022) to 1.6% (harvest in 2023). The content of phospholipids was 3.3% in the 2022 crop and 4.1% in the 2023 crop. The tocopherol content differed significantly between the two samples, with the 2023 harvest containing 2.5 times less tocopherol than the 2022 harvest. α-Tocopherol predominated in both harvests, with the 2022 crop being richer in this component compared to the 2023 crop. Additionally, γ-tocopherol was identified only in the 2022 harvest. The variation in tocopherol content may be attributed to different climatic conditions during the growth stage.

Many factors can influence the fatty acid composition of vegetable oils, including the climatic conditions, geographic regions, extraction methods, etc. The fatty acid composition of *A. tabernaemontana* seed oil was determined by gas chromatographic analysis, revealing the presence of sixteen fatty acids (Table 4).

The analysis revealed that polyunsaturated fatty acids (PUFAs) predominated (at over 60.0%), followed by monounsaturated fatty acids (MUFAs), at approximately 30.0%. The amount of saturated fatty acids (SFAs) was the lowest, constituting only about 8%. It was evident that there were no significant differences in the total SFA and UFA results of the seed oils during the examined period.

Among the PUFAs, linoleic acid (C_18:2_) was the main fatty acid, with its amount in the 2022 harvest being comparable to that in the 2023 harvest (61.0% vs. 61.2%). The content of oleic acid (C_18:1_) was also found to be equal in both crops (28.7% and 28.6%, respectively). The amount of saturated palmitic acid (C_16:0_) was close to 6%, and the content of stearic acid was below 2% in both analyzed crops. All other identified fatty acids were present in insignificant amounts (below 1%) in the seed oil of *A. tabernaemontana*, with comparable values in both the 2022 and 2023 harvests. On the other hand, the contents of stearic and γ-linolenic acid slightly decreased in the 2023 crop, while the amounts of the other fatty acids remained practically unchanged.

The findings regarding the presence of sterols, phospholipids, and tocopherols in *A. tabernaemontana* seed oil provided a strong rationale for further analyzing their individual composition (Table 5 and Table 6).

β-Sitosterol was the predominant component of the sterol fraction, with contents of 48.6% and 45.8% in the two respective crops. The amounts of campesterol and stigmasterol were identical (25.1% and 25.2%, respectively) in the 2022 harvest. However, the amount of campesterol was significantly higher in the 2023 crop (32.9%) at the expense of lower levels of stigmasterol (19.5%). A considerably smaller amount of Δ^5^-avenasterol was observed (1.1% vs. 0.7%). Additionally, small amounts of cholesterol (0.4%), Δ^7^-campesterol (0.5%), and Δ^7^-avenasterol (0.2%) were identified in the seed oil from the 2023 harvest, which were not present in the 2022 crop.

The phospholipid fraction was composed of five classes: phosphatidylcholine (PC), phosphatidylinositol (PI), phosphatidylethanolamine (PEA), phosphatidic acid (PA), and lysophosphatidylcholine (LPC). The detailed results are provided in Table 6.

The contents of identified phospholipids in the analyzed samples were evenly distributed. Phosphatidylcholine was the most abundant in both crops (33.1% vs. 30.8%), followed by phosphatidylinositol (30.5% vs. 28.9%) and phosphatidylethanolamine (19.6% vs. 17.8%). The amounts significantly differed between the 2022 and 2023 harvests, with the most notable difference observed in the content of phosphatidic acid.

## 3. Discussion

The proximate composition of the seeds gives information about their nutritional value, revealing the contents of the main nutrients present in the plant material. Members of the Apocynaceae family have been proven to be rich in alkaloids, terpenoids, steroids, flavonoids, glycosides, simple phenols, lactones, and hydrocarbons [13]. The glyceride oil, protein, carbohydrate, crude fiber, ash, and moisture contents had not previously been determined, as these components are not directly related to the medicinal properties of *A. tabernaemontana*. The content of glyceride oil in the examined seeds of *A. tabernaemontana* was significantly lower than those reported for other plant species in the Apocynaceae family, where the seed oil content ranged from 11.3% in *Mandevilla laxa* to 43.2% in *Thevetia peruviana* [14]. Typically, oily seeds and nuts, such as sunflower seeds and pine nuts, have a glyceride oil content exceeding 50.0%. Proteins are the other very important nutrients in the seeds, which have important functions, such as being structural components of the cell walls and membrane [15]. On the other hand, carbohydrates not only have structural functions but also act as a major energy reserve for seeds, protecting them from oxidative stress during storage and germination [15]. Dietary fibers are part of the carbohydrates that play a crucial role in maintaining digestive health, supporting regular bowel movements, and promoting a healthy gut microbiome. The results on the content of crude fiber align with previous reports on other medicinal plants, such as *Corchorus trilocularis* (17.46%) and *Centella asiatica* (21.78%), as reported by Vishwakarma and Dubey [16].

The physicochemical characteristics of oils can indicate either the initiation of an oxidation process or the presence of impurities in the lipids. The iodine value of the *A. tabernaemontana* seed oil is consistent with the values of soybean (124–139 g I_2_/100 g) and sunflower (118–141 g I_2_/100 g) seed oils, which are widely used as semi-dry oils [17]. The results on the refractive index are similar to that reported for *V. minor*, which was found to be 1.462 [11].

Glyceride oils primarily comprise triacylglycerols, which are fatty acids esterified with glycerol. Additionally, they contain minor components, such as unsaponifiable substances (including sterols, tocopherols, carotenoids, etc.) and polar lipids, like phospholipids and sphingolipids. The total sterol content of the *A. tabernaemontana* seed oil was comparable in both harvests (1.4% vs. 1.6%), which is relatively high compared to the sterol content in widely used vegetable oils, such as sunflower (0.24–0.46%), soybean (0.18–0.41%), and cottonseed (0.27–0.64%) [17]. Phospholipids are essential for the formation and functionality of cell membranes. Some of them act as lipid mediators of inflammation, influencing immune responses at the cellular level. Additionally, specific phospholipids and their metabolites function as secondary messengers in cellular signaling pathways [18]. There is some evidence that tocopherols possess several properties, such as anti-obesity, anti-atherosclerotic, anti-inflammatory, antihypertensive, anti-lipidemic, anti-cancer, anti-cardiovascular disease, and anti-diabetic, and can improve immune functions. Furthermore, recent studies have indicated that γ-tocopherol showed great potential in preventing diseases linked to acute inflammation and oxidative damage. Nonetheless, large-scale clinical trials are essential to validate their biological functions [19].

Fatty acids are key components of glyceride oils and can be categorized as essential and nonessential. The essential fatty acids, particularly n-3 and n-6 fatty acids, are vital for brain development and function. They contribute to cognitive performance and may help protect against neurodegenerative diseases [15]. The major fatty acids found in the *A. tabernaemontana* seed oil were linoleic, followed by oleic and palmitic. The current results on the predominance of linoleic acid in the glyceride oil of the examined samples differ from those reported for *V. minor*, where the content of this fatty acid was found to be 12.45% [10]. Another study reported amounts of linoleic acid in plants within the Apocynaceae family to be close (*Apocynum cannabinum*—58.5% and *Strophanthus speciosus*—58.7%) and sometimes exceeding our results (*Mandevilla laxa*—68.2%) [14]. In *V. minor* seed oil, the level of oleic acid (48.9%) was higher than in our findings, while the plant *Pachypodium rosulatum* (elephant’s foot plant) showed a value close to ours (27.1%) [10,14]. Other studies have shown that the amount of palmitic acid in other Apocynaceae family plants can vary widely, from 6.0% in *Asclepias incarnata* (swamp milkweed) to 29.1% in *Pachypodium rosulatum*. The amount of stearic acid ranged from 2.2% in *Asclepias incarnata* to 17.3% in *Adenium obesum* (desert rose) [14]. Extensive research has linked essential fatty acids to numerous health benefits, including reduced risks of cardiovascular diseases, enhanced brain and vision functions, cancer prevention, support for infant development, as well as improved outcomes in hypertension, arthritis, diabetes mellitus, and various neurological and neuropsychiatric conditions [20].

Sterols are one of the main components of the unsaponifiable matter. There was limited previous information regarding the quantitative and qualitative sterol composition of the seed oil in certain plants in the Apocynaceae family. Nonetheless, the qualitative sterol composition of the *A. tabernaemontana* seed oil was similar to that of the well-known palm kernel oil, of which β-sitosterol is a major component, followed by stigmasterol and campesterol [17]. The substantial amount of β-sitosterol in *A. tabernaemontana* supports its use as a medicinal plant. It is non-toxic and offers various pharmacological benefits, including anticancer (effective against breast, prostate, colon, lung, stomach, leukemia, etc.), antioxidant, anti-inflammatory, antimicrobial, immunomodulating, and antidiabetic effects. It can positively impact the cardiovascular system by preventing heart attacks and atherosclerosis [21,22,23,24]. Conversely, stigmasterol is linked to antibacterial activity against *Escherichia coli*, *Staphylococcus aureus*, *Pseudomonas aeruginosa*, and *Salmonella typhimurium* [25].

Phospholipids are a crucial component of biologically active substances due to their ability to form complexes with plant extracts or phytoactive compounds. These complexes enhance drug solubility, facilitate body penetration, and ensure sustained release. One significant advantage of phospholipid complexes is their biocompatibility, particularly with phosphatidylcholine as the main component [26]. *A. tabernaemontana* can be a source of natural phospholipids, which are preferred in pharmaceutical formulations (emulsions, suspensions, mixed micelles, and liposomal preparations) over synthetic ones. These also lead to consistent quality and lower production costs of the product [27].

## 4. Materials and Methods

### 4.1. Plant Material

The seeds of *Amsonia tabernaemontana* Walt. were collected from 10 select plants that formed a significant aerial mass. These plants were cultivated under greenhouse conditions at the Institute of Roses and Aromatic Plants and are part of the Institute’s Gene Fund (geographic coordinates Lat. 42.63430; Lon. 25.38882). The analyzed specimens were harvested in 2022 and 2023. The species were authenticated by I. Semerdjieva, a botanist at Agricultural University—Plovdiv, and voucher specimens (voucher SOM 179355) were deposited in the herbarium of Institute of Biodiversity and Ecosystem Research at the Bulgarian Academy of Sciences.

### 4.2. Chemical Composition

The lipids were extracted from the seeds with hexane by a Soxhlet apparatus for 8 h [28]. The solvent was evaporated, and the residual hexane was removed under a stream of nitrogen.

The protein content was determined using a Kjeldahl apparatus (Velp Scientifica Srl, Via Stazione, Italy) after the sample was mineralized for 35 min at 420 °C in the presence of H_2_SO_4_:H_2_O_2_ (2:1, *v*/*v*) and a catalyst. The solution was distilled in UDK 127 (Velp Scientifica Srl, Via Stazione, Italy) [29].

The carbohydrate content was calculated by FAO [30]:

100 − (weight in grams [protein + lipids + water + ash] in 100 g).

The fibers, ash content, and moisture were determined gravimetrically according to AOAC [29].

The energy value (EV) in kcal/100 g was calculated as follows:

EV = % proteins × 4.0 + % carbohydrates × 4.0 + % lipids × 9.0.

### 4.3. Physicochemical Properties

The physicochemical characteristics of the glyceride oil (peroxide value, iodine value, and refractive index) were analyzed following the procedures by ISO [31,32,33].

### 4.4. Fatty Acid Composition

The fatty acid composition of the seed oil was gas chromatographically (GC) determined [34]. The first step was transesterification with sulfuric acid in methanol of the oil to obtain the fatty acid methyl esters (FAMEs) [35]. Determination of the FAMEs was performed on an Agilent 8860 (Santa Clara, CA, USA) equipped with a flame ionization detector (FID) and capillary column (DB-Fast FAME, Agilent J&W) with the following characteristics: 30 m × 0.25 mm × 0.25 μm (film thickness). The work conditions were as follows: the column temperature was 70 °C (for 1 min), which was increased up to 250 °C at a rate of 5 °C/min (for 3 min); the temperature of the injector was 270 °C, and that of the detector was 300 °C. Identification was performed by comparison of the retention times with the retention time of a standard mixture of FAME (Supelco, Bellefonte, PA, USA 37 comp. FAME mix), which was subjected to GC analysis under identical conditions.

### 4.5. Determination of Sterols

After saponification of the seed oil with 2 N KOH, the unsaponifiable matter was extracted with hexane. Its content was determined gravimetrically [36]. Thin-layer chromatography was used for the isolation of the sterols from the unsaponifiable matter. According to Ivanov et al. [37], their content was determined spectrophotometrically at 597 nm. The sterol composition was determined on an HP 5890 gas chromatograph equipped with DB 5 capillary column (25 m 0.25 mm 0.25 µm (film thickness)) and FID. The operating conditions were as follows: the starting temperature was 90 °C (for 3 min), which was increased to 290 °C at a rate of 15 °C/min, and then increased to 300 °C at a rate of 4 °C/min (and held for 10 min). The detector temperature was 320 °C, and the injector temperature was 300 °C, with hydrogen as the carrier gas. The individual composition of the identified sterols was determined by a comparison of the retention times with a standard mixture of sterols [38].

### 4.6. Determination of Tocopherols

High-performance liquid chromatography (HPLC) was used for the determination of the content of the tocopherols. The apparatus was equipped with fluorescent detection (295 nm of excitement and 330 nm of emission) and Nucleosil Si 50-5 column (250 mm × 4 mm). The mobile phase was hexane/dioxane, 96:4 (*v*/*v*), and the flow rate was 1 mL/min. The oil was diluted with *n*-hexane to obtain a 2% solution, and 20 μL was injected into the system [39]. The total tocopherol content was calculated at the base of the peak areas in the sample vs. the peak area of a standard tocopherol solution.

### 4.7. Determination of Phospholipids

Two-dimensional thin-layer chromatography was used to isolate the phospholipid classes [40]. The spots of individual phospholipids were mineralized with a mixture of perchloric and sulfuric acid, 1:1 (*v*/*v*). Subsequently, their content was determined spectrophotometrically at 700 nm [41].

### 4.8. Statistical Analysis

The results are presented as the mean values of three parallel determinations ± standard deviations and were subjected to one-way ANOVA and compared with the Duncan test (*p* < 0.05).

## 5. Conclusions

The analysis of the chemical composition and lipid-soluble components highlights the potential of *A. tabernaemontana* as a promising alternative medicinal plant. The results of this research indicate that the peroxide value, total sterol content, and fatty acid composition of the studied glyceride oils exhibited relative stability in the second year, maintaining consistent results across both harvests. These findings underscore the robustness of these components during the investigated period.

However, it is noteworthy that significant variations were observed in the proximate composition, especially concerning the levels of tocopherol and phospholipids in the seed oils over two consecutive years. These fluctuations suggest potential factors influencing the biosynthesis or degradation of these components, warranting further investigation to fully understand their underlying mechanisms.

Moreover, the consistent presence of lipid compounds in *A. tabernaemontana* emphasizes its resilience and adaptability, making it a valuable candidate for further phytochemical and pharmacological studies.

In light of these findings, *A. tabernaemontana* emerges as a resilient and versatile species with significant medicinal potential. Continued research and exploration of this plant could lead to a deeper understanding of its role in traditional and alternative medicine.

## Figures and Tables

**Table 1:** Chemical composition of *A. tabernaemontana* seeds ^1^.

Compounds, %	Harvest 2022	Harvest 2023
Glyceride oil	7.8 ± 0.1 ^a^	11.1 ± 0.2 ^b^
Protein	19.5 ± 0.4 ^a^	13.0 ± 0.2 ^b^
Carbohydrates	60.4 ± 0.5 ^a^	61.3 ± 0.4 ^a^
Crude fiber	18.3 ± 0.1 ^a^	24.8 ± 0.3 ^b^
Ash	3.7 ± 0.1 ^a^	7.4 ± 0.1 ^b^
Moisture	8.6 ± 0.2 ^a^	7.2 ± 0.1 ^b^
Energy value, kcal/100 g	390	397

^1^ The results are presented as the mean values with the standard deviation (SD). Different small letters in a row depict significant differences in the results (*p* < 0.05).

**Table 2:** Physicochemical characteristics of *A. tabernaemontana* seed oil ^1^.

Physicochemical Characteristics	Harvest 2022	Harvest 2023
Peroxide value, meqO_2_/kg	5.45 ± 0.10 ^a^	5.29 ± 0.08 ^a^
Iodine value, g I_2_/100 g	139.8 ± 0.2 ^a^	134.4 ± 0.3 ^b^
Refractive index	1.4825 ± 0.0002 ^a^	1.4873 ± 0.0001 ^b^

^1^ The results are presented as the mean values with standard deviation (SD). Different small letters in a row depict significant differences in the results (*p* < 0.05).

**Table 3:** Content of lipid-soluble components of *A. tabernaemontana* seed oil ^1^.

Lipid-Soluble Components	Harvest 2022	Harvest 2023
Unsaponifiable matter, %	25.5 ± 0.2 ^a^	19.5± 0.2 ^b^
Sterols, %	1.4 ± 0.1 ^a^	1.6 ± 0.1 ^a^
Phospholipids, %	3.3 ± 0.1 ^a^	4.1±0.1 ^b^
Tocopherols, mg/kg	52.7 ± 0.8 ^a^	20.0 ± 0.3 ^b^
- α-Tocopherol, mg/kg	34.5 ± 0.5 ^a^	20.0 ± 0.3 ^b^
- γ-Tocopherol, mg/kg	18.2 ± 0.3	n.d.

^1^ The results are presented as the mean values with standard deviation (SD). Different small letters in a row depict significant differences in the results (*p* < 0.05); n.d.—not detected.

**Table 4:** Fatty acid composition of *A. tabernaemontana* seed oil ^1^.

Fatty Acids, %		Harvest 2022	Harvest 2023
С_8:0_	Caprylic	0.1 ± 0.0 ^a^	0.1 ± 0.0 ^a^
С_14:0_	Myristic	0.1 ± 0.0 ^a^	0.1 ± 0.0 ^a^
С_15:1_	Pentadecenoic	0.1 ± 0.0 ^a^	0.1 ± 0.0 ^a^
С_16:0_	Palmitic	5.8 ± 0.1 ^a^	6.1 ± 0.1 ^a^
С_16:1_	Palmitoleic	0.1 ± 0.0 ^a^	0.1 ± 0.0 ^a^
С_17:0_	Margaric	0.2 ± 0.0 ^a^	0.2 ± 0.0 ^a^
С_17:1_	Heptadecenoic	0.2 ± 0.0 ^a^	0.2 ± 0.0 ^a^
С_18:0_	Stearic	1.7 ± 0.1 ^a^	1.4 ± 0.0 ^b^
С_18:1_	Oleic	28.7 ± 0.2 ^a^	28.6 ± 0.1 ^a^
С_18:2_	Linoleic	61.0 ± 0.2 ^a^	61.2 ± 0.4 ^a^
С_18:3 n-6_	γ-Linolenic	0.5 ± 0.0 ^a^	0.3 ± 0.0 ^b^
С_18:3 n-3_	α-Linolenic	0.7 ± 0.1 ^a^	0.7 ± 0.0 ^a^
С_20:0_	Arachidic	0.1 ± 0.0 ^a^	0.1 ± 0.0 ^a^
С_20:1_	Eicosenoic	0.5 ± 0.0 ^a^	0.5 ± 0.0 ^a^
С_20:3 n-6_	Dihomo-γ-linolenic	0.1 ± 0.0 ^a^	0.1 ± 0.0 ^a^
С_22:0_	Behenic	0.1 ± 0.0 ^a^	0.2 ± 0.0 ^b^
Monounsaturated fatty acids		29.6 ^a^	29.5 ^a^
Polyunsaturated fatty acids		62.3 ^a^	62.3 ^a^
Saturated fatty acids		8.1 ^a^	8.2 ^a^

^1^ The results are presented as the mean values with standard deviation (SD). Different small letters in a row depict significant differences in the results (*p* < 0.05).

**Table 5:** Individual sterol composition of *A. tabernaemontana* seed oil ^1^.

Individual Sterol Composition	Harvest 2022	Harvest 2023
Cholesterol	n.d.	0.4 ± 0.0
Campesterol	25.1 ± 0.1 ^a^	32.9 ± 0.7 ^b^
Stigmasterol	25.2 ± 0.1 ^a^	19.5 ± 0.2 ^b^
Δ^7^-Campesterol	n.d.	0.5 ± 0.1
β-Sitosterol	48.6 ± 0.4 ^a^	45.8 ± 1.0 ^b^
Δ^5^-Avenasterol	1.1 ± 0.1 ^a^	0.7 ± 0.1 ^b^
Δ^7^-Avenasterol	n.d.	0.2 ± 0.0

^1^ The results are presented as the mean values with standard deviation (SD). Different small letters in a row depict significant differences in the results (*p* < 0.05); n.d.—not detected.

**Table 6:** Phospholipid composition of *A. tabernaemontana* seed oil ^1^.

Phospholipids, %	Harvest 2022	Harvest 2023
Phosphatidylcholine	33.1 ± 0.2 ^a^	30.8 ± 0.1 ^b^
Phosphatidylinositol	30.5 ± 0.2 ^a^	28.9 ± 0.2 ^b^
Phosphatidylethanolamine	19.6 ± 0.1 ^a^	17.8 ± 0.1 ^b^
Phosphatidic acids	8.7 ± 0.1 ^a^	7.7 ± 0.2 ^b^
Lysophosphatidylcholine	8.1 ± 0.2 ^a^	14.8 ± 0.3 ^b^

^1^ The results are presented as the mean values with standard deviation (SD). Different small letters in a row depict significant differences in the results (*p* < 0.05).

## Data Availability

The data presented in this study are available upon request from the corresponding author.
